# C-952-01. Artificial Intelligence in Burn Assessment: A Systematic Review of Clinical Performance

**DOI:** 10.1093/jbcr/irag033.117

**Published:** 2026-04-06

**Authors:** Natalie Carter, Christopher J Fedor, Bilal M Chaudhry, Jenny A Ziembicki, Francesco M Egro

**Affiliations:** Herbert Wertheim College of Medicine, Westchester, FL; University of Pittsburgh Medical Center, Department of Plastic Surgery, Pittsburgh, PA; Rutgers University, Newark, NJ; University of Pittsburgh Medical Center, Pittsburgh, PA; University of Pittsburgh Medical Center, Pittsburgh, PA

## Abstract

**Introduction:**

Accurate burn assessment is essential for guiding resuscitation, surgical decision-making, and triage, yet clinical evaluation of total body surface area (TBSA) and burn depth remains highly subjective. Even among trained clinicians, misclassification can lead to under- or over-resuscitation, delayed treatment, and inappropriate operative planning. Artificial intelligence (AI) has emerged as a promising tool to provide objective and reproducible assessment. While earlier work relied on handcrafted features and classical machine learning, more recent convolutional neural networks (CNNs), hybrid architectures, and ensemble approaches have demonstrated markedly higher performance by directly learning image features and capturing pixel-level detail.

**Methods:**

We systematically reviewed 24 published studies applying AI to burn assessment, spanning three major domains: TBSA estimation, tissue classification, and wound differentiation/detection. Extracted data included model type, dataset characteristics, ground truth definition, and reported performance metrics such as accuracy, precision, sensitivity, specificity, F1-score, and, where available, area under the ROC curve (AUC).

**Results:**

Across all tasks, AI models demonstrated strong and often superior performance. For TBSA estimation, U-Net, Mask R-CNN, and hybrid DenseMask RCNN achieved accuracies up to 0.92, with mean F1-scores of 0.82 and high specificity (0.93), supporting reliable delineation of burn areas. Tissue classification studies reported accuracies as high as 0.96, with CNNs and attention-based architectures consistently outperforming traditional methods; ensemble approaches further improved results. Wound differentiation and detection yielded the highest performance overall, with AUCs ranging from 0.91 to 0.99 (mean 0.94), accuracies averaging 0.92, and F1-scores near 0.96, underscoring excellent discriminative ability across burns, ulcers, bruises, and normal skin.

**Conclusions:**

Reported accuracies for AI in burn assessment now consistently exceed 85-90%, with AUCs approaching 0.99 in several domains, suggesting that these systems are nearing the reliability needed for clinical application. Pixel-level annotations and hybrid models appear to drive the strongest outcomes. However, heterogeneity in reported metrics and study designs limits comparability and highlights the need for standardized benchmarks, larger datasets, and prospective validation before integration into routine care.

**Applicability of Research to Practice:**

AI-based burn assessment tools hold particular promise for triage, telemedicine, and operative decision-making, especially in hospitals without specialized burn centers. As the technology matures, the central challenge will be translating high-performance models into robust, generalizable systems capable of supporting clinicians in real-world settings.

**Funding for the study:**

N/A.

